# The Obesity Risk SNP (rs17782313) near the MC4R Gene Is Not Associated with Brain Glucose Uptake during Insulin Clamp—A Study in Finns

**DOI:** 10.3390/jcm10061312

**Published:** 2021-03-23

**Authors:** Eleni Rebelos, Miikka-Juhani Honka, Laura Ekblad, Marco Bucci, Jarna C. Hannukainen, Lilian Fernandes Silva, Kirsi A. Virtanen, Lauri Nummenmaa, Pirjo Nuutila

**Affiliations:** 1 Turku PET Centre, University of Turku, 20520 Turku, Finland; llekbl@utu.fi (L.E.); marbuc@utu.fi (M.B.); jhannukainen@gmail.com (J.C.H.); kianvi@utu.fi (K.A.V.); latanu@utu.fi (L.N.); 2 Turku PET Centre, Turku University Hospital, 20521 Turku, Finland; miikka.honka@tyks.fi; 3 Division of Clinical Geriatrics, Center for Alzheimer Research, Department of Neurobiology, Care Sciences and Society, Karolinska Institutet, 17177 Stockholm, Sweden; 4 Institute of Clinical Medicine, Internal Medicine, University of Eastern Finland, 70210 Kuopio, Finland; lilian.fernandes.silva@uef.fi; 5 Department of Psychology, University of Turku, 20520 Turku, Finland; 6 Department of Endocrinology, Turku University Hospital, 20521 Turku, Finland

**Keywords:** single nucleotide polymorphism, rs17782313, positron emission tomography, insulin resistance, brain glucose uptake

## Abstract

The melanocortin system is involved in the control of adiposity through modulation of food intake and energy expenditure. The single nucleotide polymorphism (SNP) rs17782313 near the *MC4R* gene has been linked to obesity, and a previous study using magnetoencephalography has shown that carriers of the mutant allele have decreased cerebrocortical response to insulin. Thus, in this study, we addressed whether rs17782313 associates with brain glucose uptake (BGU). Here, [^18^F]-fluorodeoxyglucose positron emission tomography (PET) data from 113 Finnish subjects scanned under insulin clamp conditions who also had the rs17782313 determined were compiled from a single-center cohort. BGU was quantified by the fractional uptake rate. Statistical analysis was performed with statistical parametric mapping. There was no difference in age, BMI, and insulin sensitivity as indexed by the M value between the rs17782313-C allele carriers and non-carriers. Brain glucose uptake during insulin clamp was not different by gene allele, and it correlated with the M value, in both the rs17782313-C allele carriers and non-carriers. The obesity risk SNP rs17782313 near the *MC4R* gene is not associated with brain glucose uptake during insulin clamp in humans, and this frequent mutation cannot explain the enhanced brain glucose metabolic rates in insulin resistance.

## 1. Introduction

Obesity represents a major health burden, considering that the number of obese individuals worldwide is approximately 650 million. Despite intensive research, the current pathophysiology of obesity is still incompletely understood. Gene mutations have been shown to associate only with a minority of obesity cases (<3%), and genetic variation that leads to obesity mainly involves genes expressed in the central nervous system (CNS) [1]. Melanocortin 4 receptor (*MC4R*) deficiency is the commonest monogenic form of obesity, and results in severe obesity [2]. A much more common genetic variant rs17782313 (C/T polymorphism) near the *MC4R* gene has also been associated with obesity, and insulin resistance [3]. The frequency of the C allele has been described to vary from 13% to 40% across the major populations [4,5]. Genome-wide association studies have shown that carriers of the minor C allele are at an increased risk for obesity, when compared to carriers of the more common form T, and that the risk for obesity is further increased in CC homozygotes [3]. 

The melanocortin receptors (MCR), mainly situated in the ventromedial hypothalamus, receive information from proopiomelanocortin (POMC) and agouti-related protein (AgRP) neurons in the arcuate nucleus regarding the nutritional status of the body. POMC is cleaved by prohormone convertases to yield α-melanocyte-stimulating hormone [6], which activates the melanocortin receptors to decrease food intake, whereas AgRP antagonizes *MC4R*. Recently, Tschritter and colleagues have shown that carriers of the rs17782313-C allele had blunted cerebrocortical response to insulin in magnetoencephalography studies (MEG) [7]. Based on this finding, the authors proposed that the rs17782313-C allele is linked to brain insulin resistance. 

However, until today, there is no common understanding for the definition of brain insulin resistance. Whereas peripheral insulin resistance has been defined based on tissue-level studies, where several molecular defects have been established, the definition of central insulin resistance has been to some extent arbitrary, and it has majorly depended on which of the different neuroimaging approaches were used to study it. Consequently, a blunted cerebrocortical response to insulin (MEG), and a decreased suppression of hypothalamic blood flow (fMRI) have been defined as central insulin resistance [8,9]. Our group on the contrary, has consistently found increased brain glucose uptake (BGU) during conditions of euglycemic insulin clamp in obese/insulin resistant individuals [10], and also in individuals carrying a loss-of-function variant of the gene AKT2 that increases the risk for type 2 diabetes [11]. In a recent report, we have tried to underline the problem of lack of a ground truth definition of central insulin resistance, and the need for multiple measurements with different neuroimaging modalities in the same subjects so that the findings of neuroimaging studies can be better understood [12]. In this study, based on the previous findings of Tschritter and colleagues, we wished to evaluate whether in our dataset of brain insulin clamp [^18^F]FDG/PET studies, presence of the rs17782313-C allele would associate with increased BGU during insulin stimulation. 

## 2. Materials and Methods

Study population: We determined rs17782313 in the *MC4R* gene in 113 Finnish subjects who have previously participated in [^18^F]-FDG-PET studies during euglycemic hyperinsulinemia [13]. Eighteen percent were morbidly obese subjects studied before undergoing bariatric surgery. Fifteen percent were subjects with type 2 diabetes or impaired fasting glucose/impaired glucose tolerance and six percent were overweight individuals. Sixty one percent were healthy controls (normal BMI, absence of type 2 diabetes, dyslipidemia or hypertension, and normal biochemical results including renal function and transaminases). None had a clinical diagnosis of neurological diseases. Patients with type 2 diabetes used either metformin (1–3 g daily), or combination of metformin and dipeptidyl peptidase-4 inhibitors. Patients on insulin treatment were excluded. All subjects underwent a screening visit before inclusion in the study. Metformin was withheld 24–72 h and dipeptidyl peptidase-4 inhibitors 24 h before the metabolic study. Prior to inclusion, each participant gave a written consent. Each study protocol included in this study was approved by the Ethics Committee of the Hospital District of Southwest Finland and conducted in accordance with the Declaration of Helsinki. 

Euglycemic hyperinsulinemic clamp [^18^F]-FDG studies: The euglycemic hyperinsulinemic clamp was performed as previously described. In brief, a primed-continuous infusion of insulin (Actrapid, Novo Nordisk, Copenhagen, Denmark) was given at a rate of 40 mU·m^−2^·min^−1^. During the clamp, a variable rate 20% glucose solution was infused to maintain euglycemia at ~5 mmol/L. Plasma glucose levels were measured every 5–10 min throughout the clamp. At 100 ± 10 min into the clamp, [^18^F]-FDG (187 ± 9 MBq) was injected intravenously over 15 s and the acquisition of brain radioactivity started either immediately afterward (n = 54) or ~1 h after [^18^F]-FDG injection (n = 59). The PET protocols are described in more detail in previous reports [10]. During the clamp, samples for plasma insulin and serum FFA measurement were taken at baseline and at 30 and 60 min, respectively thereafter.

Quantification of brain glucose uptake: Brain glucose uptake (BGU, in μmol·100 g^−1^·min^−1^) was calculated at voxel level as fractional uptake rate (FUR) times average plasma glucose concentration from the injection till the end of the brain scan, divided by the lumped constant for the brain (which was set at 0.65) [14]. For the early scans, the FUR calculation was restricted between 30 and 40 min. For the late scans, all frames were included.

Calculation of insulin-stimulated glucose disposal (M value): M value was calculated as a measure of whole-body insulin sensitivity, as previously described and expressed per kg of fat-free mass (μmol·kg_FFM_^−1^·min^−1^), as this normalization has been shown to minimize differences due to sex, age, and body weight [15]. Fat-free mass was estimated using the allometric equation of Watson et al. [16]. 

Genotyping: DNA was isolated from the whole blood. Genotyping was done using Illumina Human OmniExpress analysis Bead Chip at the Institute for Molecular Medicine, Helsinki, Finland.

Statistical analysis: Data are presented as mean ± SD (or median (IQR) for non-normally distributed variables). Categorical variables are presented as absolute numbers. Continues variables were compared using the Student’s *t*-test or nonparametric Mann–Whitney *U*-test, as appropriate. Categorical variables were compared using a Chi-square test. Comparisons between groups were performed in SPM with a two-sample independent t tests, while controlling for confounding factors (setting 0 in the contrasts). The statistical threshold in SPM analysis was set at a cluster level and corrected with false discovery rate (FDR) with *p* < 0.05. Further statistical analyses were done using JMP version 13.0 (SAS Institute, Cary, NC, USA).

Statistical power: Power analysis was not performed a priori as our study was done using pre-existing data. However, a power analysis using our sample size and the Cohen’s d effect size 0.67 for the difference between the genotype groups from Tschritter’s study gave 95% power when alpha was set to 5%. 

## 3. Results

The genotype distribution was TT 79, TC 30, and CC 4 (prevalence 70%, 27%, 3%, respectively). 

Because of the low number of the CC carriers, comparisons have been performed both among the three different groups and separately between TT and TC carriers only. Even though TC group had slightly higher BMI and worse insulin sensitivity as indexed by the M value compared to the TT group, we found no statistically significant differences in the parameters evaluated comprising waist and waist/hip ratio, blood pressure, HBA_1c_, and cholesterol values (Table 1). 

### Brain Glucose Uptake and rs17782313 Allele

**Figure 1:**
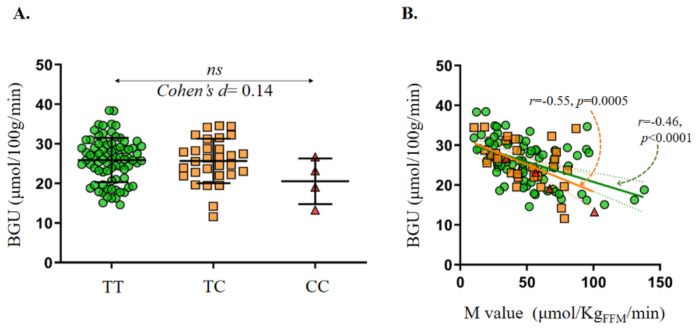
No difference in BGU during insulin stimulation was found between the subjects homozygous or heterozygous for rs17782313-C allele as compared to the carriers of the TT genotype (**A**). Clamp BGU correlated negatively to the M value in both the subjects carrying the rs17782313-C allele and the carriers of the TT genotype (**B**). In 1A mean ± SD is given. In 1B the orange line represents the slope and 95% Confidence interval for the grouped TC/CC subjects.

In the whole dataset, there was no evidence of higher BGU during clamp in the rs17782313-C allele carriers as compared to the TT genotype carriers, a finding which was also confirmed at the whole-brain, voxel-by-voxel analysis with SPM (Figure 1A). Also, in a multivariate analysis including age and BMI, or age and M value, or age and glucose tolerance status, presence of the C-allele had not an independent effect on BGU. In line with previous reports clamp BGU correlated negatively with the M value, and this association was present both in the rs17782313-C allele carriers and the TT genotype carriers (Figure 1B). 

In a subset of 44 subjects who had BGU measured both in conditions of fasting and insulin clamp, there was still no difference in δBGU, or in fasting BGU between the two groups. 

## 4. Discussion

The main finding of the present study among Finns was that presence of the obesity risk allele rs17782313-C does not affect brain glucose uptake in conditions of insulin clamp, or in a smaller set of subjects studied both under fasting and clamp conditions. Of note, the rs17782313-C allele carriers in our study did not have worse systemic insulin sensitivity evaluated with the M value of the insulin clamp, or higher adiposity measurements. 

In a previous study the Tübingen group has reported that carriers of the obesity risk allele rs17782313-C had blunted insulin-stimulated cerebrocortical theta activity compared to non-carriers even though there was no difference in the insulin sensitivity index (M value) in the hyperinsulinemic euglygemic clamp between the groups [7]. Based on these findings the authors proposed that cerebral insulin resistance may contribute to the obesity effect of rs17782313-C. However, cerebral insulin resistance is a demanding topic to address in humans, and previous neuroimaging studies have defined central insulin resistance according to the neuroimaging method used. More particularly, a blunted cerebrocortical response to insulin when MEG is applied [7], or a decreased suppression of hypothalamic blood flow after intranasal insulin administration in fMRI [9] have both been defined as cerebral (or central) insulin resistance. Our group and others have consistently reported increased metabolic rates in obese/insulin resistant subjects in [^18^F]-FDG-PET studies, under conditions of euglycemic hyperinsulinemia [17,18,19]. Of note, MEG measures net effect of the ionic currents in neurons during synaptic transmission, BOLD fMRI evaluates cerebral blood flow and cerebral metabolic rate of oxygen, while [^18^F]-FDG-PET quantifies glucose metabolic rates at the whole-brain level, and also in specific parts of the organ. However, little is known about the molecular basis of these findings, and to the best of our knowledge only one study has demonstrated cerebral insulin resistance, defined as an attenuated response to insulin incubation by neuronal insulin receptors, in a postmortem study in patients with Alzheimer’s disease (AD), when compared to cognitively normal individuals and patients with mild cognitive impairment [20], suggesting that cerebral insulin resistance may be a key alteration in the pathophysiology of AD. Of note, BGU is thought to be largely regulated by the insulin-independent glucose transporter, GLUT1 [21,22], and previous studies have shown that insulin has numerous actions in the central nervous system that are independent of BGU [23]. Therefore, the different methods assessing the brain’s response to insulin in vivo (during the insulin clamp or after intranasal insulin administration) most probably capture different aspects insulin action in the brain, depending on whether these methods assess insulin-dependent glucose uptake, or the effect of insulin on synaptic function or regional cerebral blood flow. 

Since there is no feasible method to evaluate brain insulin resistance in humans at tissue level, addressing brain metabolism in the living human brain and in a relatively minimally invasive manner relies on neuroimaging. Since neuroimaging studies are difficult and expensive to perform, and only few specialized neuroimaging centers exist around the globe, there is a thus far unmet need to translate the findings of one neuroimaging approach to the other [12]. For this reason, in the present study, we addressed whether in our [^18^F]-FDG/PET dataset, carrying the rs17782313-C allele could have affected the central findings, in parallel to the findings of Tschritter et al. As in the aforementioned study, also in our dataset presence of the obesity risk allele rs17782313-C did not lead to differences in BMI or insulin sensitivity (M value). The two groups were also well-matched for age. In contrast to the findings of Tschritter et al., there was no difference in BGU between mutation carriers and non-carriers, and the same was true when accounting for possible confounders, such as the M value, age, and study. We also found no evidence of an effect of the mutation in a small subset of subjects (N = 44, 11 TC and 33 TT subjects) who were studied both under fasting and clamp conditions.

The discrepancy between our results (no difference in terms of BGU) and the results of Tschritter and colleagues (blunted cerebrocortical insulin response to the rs17782313-C allele carriers), may be attributed to several factors. First and foremost, these two neuroimaging methods (MEG and [^18^F]-FDG-PET) characterize different aspects of brain function. Thus, small alterations in the hypothalamic function induced by this mutation may affect the neuronal firings that are captured by MEG, but not the brain glucose metabolism studied by [^18^F]-FDG-PET. MEG can only yield semi-quantitative indices of brain insulin sensitivity, as it measures dendritic currents and thus does not directly index brain metabolism. Second, Tschritter and colleagues studied brain insulin response by comparing the MEG response during the insulin clamp to a placebo-experiment in the same subject where a saline infusion was used instead of an insulin infusion. In the study comparing MEG response in rs17782313-C allele carriers and non-carriers the MEG data of the saline experiment was subtracted from the insulin experiment data. This method might be more sensitive in capturing individual alterations in brain insulin response than the comparison of insulin-stimulated BGU between mutation carriers and non-carriers. However, it should be noted that also in the fasting-clamp experiment (N = 44) we did not find any difference between the two groups. Third, these differences may be attributed to differences in the studied subjects. The effect of the rs17782313-C allele on eating behavior has also been shown to be sex-specific [24] and thus small differences in the sex distribution of the TC/CC allele and TT genotype carriers between our study and the study by Tschritter et al. (higher percentage of women in our study), could also explain the difference in our findings. Finally, since, in genetic studies typically larger numbers of studied subjects are needed, it cannot be excluded that our and Tschritter et al. studies suffered type 1 and type 2 statistical errors respectively, and larger number of subjects would be needed in future studies. However, according to a post hoc power analysis, the Cohen’s d effect size of 0.14 for the difference in BGU between the genotype groups observed in our study would require a future study to have over 1200 persons sample size to achieve 80% power and 5% alpha which is practically not feasible.

Our study has some further limitations. First, even though the frequency of the studied SNP is relatively similar in various populations, in this study we studied only Finnish individuals, thus the findings might not be identical in all populations. We only had 4 subjects with the CC genotype, but the prevalence of the CC genotype in our study is similar to that found in the general Finnish population [4]. Second, we combined data from several projects originally focusing on different research questions, and the data are thus not optimally balanced across different covariates. However, all data were processed and modeled with the same way to ensure consistency in the findings. Since the *MC4R* is expressed in the hypothalamus, the study of the hypothalamic glucose uptake would have been of especial interest in this report. Unfortunately, due to the physics of the PET, small brain areas such as the hypothalamus cannot be examined. Finally, it would have been of interest to evaluate whether this central mutation could relate to any adverse alimentary patterns or whether it could predict weight gain at follow-up, but these aspects were not evaluated in the present dataset. 

In conclusion, in a Finnish population sample studied, we found no effect of the common obesity risk allele rs17782313-C on brain glucose uptake in conditions of euglycemic insulin clamp, thus contrasting the previous report which addressed insulin action on the brain via MEG. Even though the differences in these two reports may be due to the different neuroimaging method used, our study underlines the fact that the definition of brain insulin resistance addressed with neuroimaging is complicated and there is currently no consensus on the ideal neuroimaging method which can address central insulin resistance. Ideally, multimodal neuroimaging studies which would use both MEG, PET, and fMRI methods in the same subjects and the same metabolic conditions could clarify which aspects of central insulin resistance these methods capture and how these results relate to each other.

## Figures and Tables

**Table 1:** Anthropometric and biochemical characteristics of the study participants by rs17782313 genotype *.

Genotype	TT	TC	CC	*p* Value	*p* Value (TT vs. TC Only)
N (%)	79 (70%)	30 (27%)	4 (3%)	-	-
Gender (F/M)	64/15	26/4	4/0	0.3	0.5
Age (years)	54 ± 15	56 ± 14	54 ± 16	0.7	0.6
BMI (Kg·m^−2^)	27.9 (10.3)	28.6 (10.5)	23.0 (4.3)	0.2	0.5
Waist (cm)	97 ± 17	98 ± 19	81 ± 11	0.2	0.7
Waist/Hip ratio	0.89 ± 0.08	0.89 ± 0.08	0.87 ± 0.08	0.7	1.0
HbA1c %	5.6 (0.5)	5.7 (0.6)	5.5 (0.3)	0.6	0.5
Fasting plasma glucose (mmol/L)	5.7 (0.9)	5.6 (0.6)	5.3 (1.7)	0.4	0.3
Fasting plasma insulin (pmol/L)	56 (63)	53 (63)	35 (42)	0.3	0.6
HOMA-IR	2.1 (1.9)	1.8 (2.4)	1.1 (1.9)	0.3	0.8
M value (μmol·kg^−1^·min^−1^)	46.9 (34.3)	43.3 (42.2)	61.8 (52.4)	0.8	
non-T2D/T2D	64/16	28/2	3/1	0.2	0.07
Total cholesterol (mmol/L)	4.8 (1.5)	4.9 (1.2)	5.5 (0.9)	0.4	0.9
HDL cholesterol (mmol/L)	1.5 (0.6)	1.5 (0.5)	2.0 (1.2)	0.4	0.6
LDL cholesterol (mmol/L)	2.6 (1.3)	2.7 (1.1)	2.6 (1.3)	0.9	0.8
Triglycerides (mmol/L)	1.0 (0.7)	0.8 (0.5)	1.0 (0.7)	0.4	0.3
Systolic BP (mmHg)	135 (31)	127 (36)	128 (75)	0.8	0.7
Diastolic BP (mmHg)	85 (15)	80 (11)	87 (17)	0.5	0.3
BGU during insulin clamp (μmol·100g^−1^·min^−1^)	25.9 ± 5.6	25.7 ± 5.6	20.6 ± 5.8	0.5	0.9

* Entries are mean ± SD, or median (interquartile range) as appropriate. T2D: type 2 diabetes.

## Data Availability

The data presented in this study are available on request from the corresponding author. The data are not publicly available due to privacy reasons.
